# Identification of Serum MicroRNAs as Novel Biomarkers in Esophageal Squamous Cell Carcinoma Using Feature Selection Algorithms

**DOI:** 10.3389/fonc.2018.00674

**Published:** 2019-01-21

**Authors:** Deqiang Zheng, Yuanjie Ding, Qing Ma, Lei Zhao, Xudong Guo, Yi Shen, Yan He, Wenqiang Wei, Fen Liu

**Affiliations:** ^1^Department of Epidemiology and Health Statistics, School of Public Health, Beijing Municipal Key Laboratory of Clinical Epidemiology, Capital Medical University, Beijing, China; ^2^National Cancer Center/Cancer Hospital, Chinese Academy of Medical Science & Peking Union Medical College, Beijing, China; ^3^Department of Molecular Physiology and Biophysics, Holden Comprehensive Cancer Center, University of Iowa Carver College of Medicine, Iowa City, IA, United States

**Keywords:** esophageal squamous cell carcinoma, serum microRNA, multiple-testing criterion, Lasso logistic regression, penalized support vector machine

## Abstract

**Introduction:** Circulating microRNAs (miRNAs) are promising molecular biomarkers for the early detection of esophageal squamous cell carcinoma (ESCC). We investigated the serum miRNA expression profiles from microarray-based technologies and evaluated the diagnostic value of serum miRNAs as potential biomarkers for ESCC by using feature selection algorithms.

**Methods:** Serum miRNA expression profiles were obtained from 52 ESCC patients and 52 age- and sex-matched controls via performing a high-throughput microarray assay. Five representative feature selection algorithms including the false discovery rate procedure, family-wise error rate procedure, Lasso logistic regression, hybrid huberized support vector machine (SVM), and SVM using the squared-error loss with the elastic-net penalty were jointly carried out to select the significantly differentially expressed miRNAs based on the miRNA profiles.

**Results:** Three miRNAs including miR-16-5p, miR-451a, and miR-574-5p were identified as the powerful biomarkers for the diagnosis of ESCC. The diagnostic accuracy of the combination of these three miRNAs was evaluated by using logistic regression and the SVM. The averages of the area under the receiver operating curve and classification accuracies based on different classifiers were more than 0.80 and 0.79, respectively. The cross-validation results suggested that the three-miRNA-based classifiers could clearly distinguish ESCC patients from healthy controls. Moreover, the classifying performance of the miRNA panel persisted in discriminating the healthy group from patients with ESCC stage I-II (AUC > 0.76) and patients with ESCC stage III-IV (AUC > 0.80).

**Conclusions:** These results in this study have moved forward the identification of novel biomarkers for the diagnosis of ESCC.

## Introduction

Esophageal cancer (EC) is the seventh most common malignancy and the sixth leading cause of cancer-related death worldwide in 2018 (1). Esophageal squamous cell carcinoma (ESCC) accounts for over 90% of all EC cases in lower income countries, especially in parts of Asia (1). Despite the improvements in surgical techniques and perioperative management have extensively prolonged the survival of ESCC patients, ESCC still remains one of the most deadly carcinomas of the gastrointestinal tract. The 5-year survival rate of late-stage ESCC is <15% (2). Therefore, early detection represents an essential way of reducing the morbidity and mortality of ESCC. Currently, diagnosis of ESCC mainly depends on endoscopic examination with biopsy, meaning that accurate detection of ESCC can only be obtained when patients have obvious symptoms and lesions. However, most early-stage ESCC patients are asymptomatic and their lesions are confined to the mucosa or submucosa, thus they usually lose the opportunity to be early diagnosed. Moreover, the invasiveness and the potential for sample error limit the effectiveness of the endoscopic biopsy (3). Thus, the discovery of novel non-invasive biomarkers with high efficiency for early detection of ESCC is urgently needed.

MicroRNAs (miRNAs) are a class of small non-coding RNAs of about 18–25 nucleotides in length. MiRNAs regulate gene expression by direct binding to the 3′ untranslated region (3′UTR) of target messenger RNAs (mRNAs) according to base pair complementarity to promote their degradation and/or translational inhibition (4). The altered expression of specific miRNAs has been associated with various diseases, including cancers (5–8). In recent years, several studies have analyzed the serum miRNA profiles in human ESCC patients by microarray-based techniques, and have examined their potential clinical relevance (9–11). However, a comprehensive evaluation of the value of circulating miRNAs as potential biomarkers for screening ESCC has yet to be investigated. Moreover, for the microarray data, the number of samples is usually much smaller than that of measured miRNAs, and this limitation is known as high-dimension, low-sample-size (HDLSS) problem that may lead to over-fitting and negatively influence the diagnostic performance in traditional statistical models. Therefore, the identification of miRNAs as biomarkers is tightly linked with the curse of dimensionality. Feature selection algorithms including multiple hypotheses testing and Lasso-type variable selection methods that have been proposed to reduce the dimension due to their simplicity and efficiency are more suitable for high-dimensional microarray data of miRNAs (12).

In this case-control study, we profiled serum miRNA expression of a relatively large number of samples using the Agilent miRNA array. The combined application of the false discovery rate (FDR) procedure (13), the family-wise error rate (FWER) procedure (14), Lasso Logistic regression (15), hybrid huberized support vector machine (HHSVM) (16)and support vector machine using the squared-error loss (SESVM) with the elastic-net penalty (17) was then carried out, and identified three serum miRNAs, including miR-16-5p, miR-451a, and miR-574-5p, as candidate biomarkers for diagnosing ESCC. We then developed three-miRNA-based classifiers based on these three miRNAs by carrying out Logistic regression and SVM model to predict ESCC. We assessed the predictive accuracies of these classifiers and discovered that they were highly efficient in discriminating ESCC patients from healthy controls, suggesting these miRNAs are potential diagnostic biomarkers for ESCC.

## Materials and Methods

### Patients and Healthy Controls

The participants were composed of 104 Chinese Han people, including 52 unrelated ESCC patients and 52 healthy controls. All ESCC cases were pathologically diagnosed for the first time and were recruited consecutively from the Endoscopy Center of Cancer Hospital, Linzhou City, Henan province, from October 2014 to October 2015. During the same period, healthy controls were randomly selected from an early EC screening program, which were frequency-matched to the ESCC cases by age (±5 years) and gender. All enrolled ESCC case and control subjects were residents in Linzhou city. This study was approved by the Institutional Review Board of Capital Medical University and was in accordance with the principle of the Declaration of Helsinki. All subjects recruited for this study provided written informed consent prior to participation. Detailed characteristics of ESCC patients and the healthy volunteers were summarized in Table 1.

**Table 1:** Characteristics of the study subjects.

**Characteristic**	**ESCC (*n* = 52)**	**Healthy (*n* = 52)**	***P***
Sex, *n* (%)			1.000
Male	24 (46.2)	24 (46.2)	
Female	28 (53.8)	28 (53.8)	
Age (years)	59.2 (4.1)	57.8 (5.5)	0.066
Smoking, *n* (%)			0.516
No	35 (67.3)	39 (75.0)	
Yes	17 (32.7)	13 (25.0)	
Drinking, *n* (%)			0.093
No	37 (71.2)	45 (86.5)	
Yes	15 (28.8)	7 (13.5)	
Stage, *n* (%)			
ESCC I-II	23 (44.2)	-	-
ESCC III-IV	29 (55.8)	-	

*ESCC I-II, ESCC with stage I-II; ESCC III-IV, ESCC with stage III-IV*.

### Total RNA Extraction

Whole blood samples were loaded into the serum collection tubes and stood for 1 h at room temperature, followed by being centrifuged at 820 g for 10 min at 4°C. The resulting serum was transferred into new tubes, followed by further centrifugation at 16, 000 g for 10 min at 4°C. Total RNA was extracted from 400 μl serum and then purified by using the mirVanaTM PARISTM kit (Ambion, Austin, TX, USA) according to the manufacturer's instructions. The quantity and purity of RNA were measured with the NanoDrop 1000 Spectrophotometer (NanoDrop Technologies, Waltham, MA, USA), and the concentration of extracted RNA ranged from 4.5 to 15.7 ng/μl. The integrity of RNA was assayed by capillary electrophoresis with an Agilent Bioanalyzer 2100 (Agilent Technologies, Santa Clara, CA, USA).

### Agilent MiRNA Microarray

The serum RNA samples from the 52 ESCC patients and 52 healthy controls were assayed for miRNA expression profiles by using the Agilent Human microRNA microarray, which contains the probes for 2006 human miRNAs. Briefly, 100 ng total RNA was labeled with Cy3 by using the miRNA Complete Labeling and Hyb Kit (Agilent Technologies, Santa Clara, CA, USA) according to the manufacturer's instructions. Each slide was hybridized with 100 ng Cy3-labeled RNA in a hybridization oven (Agilent Technologies, Santa Clara, CA, USA) at 55°C, 20 rpm for 20 h according to the manufacturer's instructions. After hybridization, slides were washed with the Gene Expression Wash Buffer Kit (Agilent Technologies, Santa Clara, CA, USA) in staining dishes (Thermo Shandon, Waltham, MA, USA). The washed slides were scanned and the microarray images were then converted into spot intensity values by the Agilent Microarray Scanner (Agilent Technologies, Santa Clara, CA, USA). The signals undergone background subtraction were exported directly into the Feature Extraction software 10.7 (Agilent Technologies, Santa Clara, CA, USA) with default settings. Raw data were normalized by the quartile algorithm with the Gene Spring Software 12.6 (Agilent Technologies, Santa Clara, CA, USA).

### Statistical Analysis

**Figure 1:**
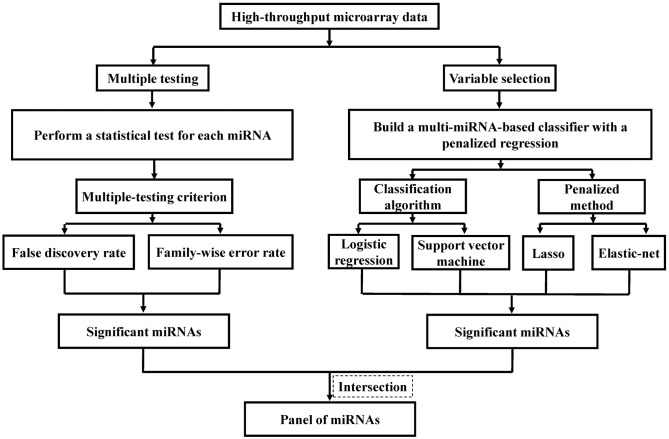
Flowchart of identifying predictive miRNAs by performing representative feature selection algorithms.

The intersection among the significant miRNAs selected by different statistical methods is identified as the candidate biomarkers for ESCC (Figure 1). For each miRNA, the non-parametric Wilcoxon rank-sum test was used to compare expression levels between the ESCC and the control group. The single *p*-value of each test is then calculated. The adjusted *p*-values are computed by the FDR method (13) and the FWER method of Holm's procedure (14), and miRNAs with an adjusted *p*-value less than or equal to 0.05 are defined significant and can be utilized as candidate biomarkers of ESCC. Simultaneously, the Lasso logistic regression (15), HHSVM (16) and SESVM (17) were performed to select the most useful diagnostic biomarkers from all ESCC associated miRNAs based on the microarray data. The intersection among significant miRNAs selected by different statistical methods is identified as a panel of biomarkers for the diagnosis of ESCC. Integrating multiple biomarkers in logistic regression and SVM, multi-miRNA classifiers are constructed to predict ESCC. The performance of the multi-miRNA classifiers is measured by classification accuracy and the area under the receiver operating characteristic (ROC) curve (AUC). Sensitivities and specificities of multi-miRNA classifiers were determined by the highest Youden index. The cross-validation was applied to evaluate the diagnostic performance. Each split of data set in cross-validation is 3/2 for training and 1/3 for testing.

The heatmap of significant miRNAs identified by the FDR method was plotted with MultiExperiment Viewer (MeV, version 4.9, TM4, Boston, MA, USA). Each significant miRNA was standardized independently by performing Z-score transformation to scale the log base 2 of the expression levels into having a mean of zero and standard deviation of one. All statistical analysis was performed with R software (version 3.4.1, R Foundation for Statistical Computing, Vienna, Austria). The R packages “glmnet” and “gcdnet” were used to implement the biomarker screening by Lasso logistic regression, HHSVM, and SESVM. The package “e1071” was used to perform SVM, and the package “pROC” was employed to plot the ROC curve and to determine the AUC.

## Results

### Identification of Significant MiRNAs by Controlling FDR and FWER

**Figure 2:**
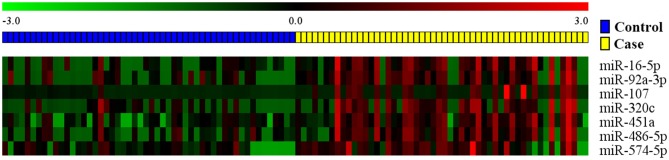
The heat map of the seven candidate miRNAs identified by the FDR method. Pseudocolors indicated expression levels on a log-2 scale from −3 to 3 standard deviations independently by Z-score transformation. Green: negative, under-expressed; red: positive, over-expressed.

**Figure 3:**
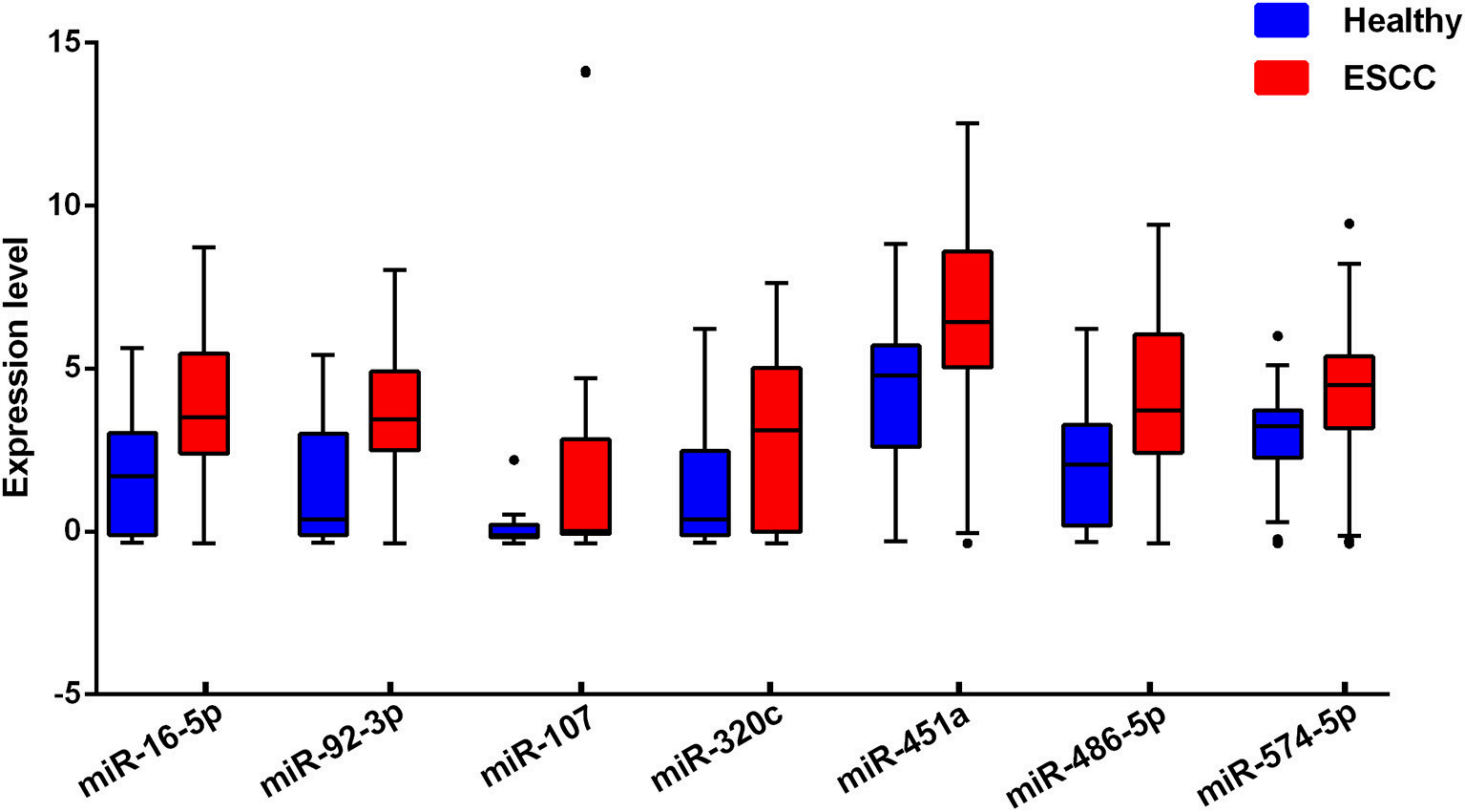
Serum levels of the seven selected miRNAs in ESCC cases and healthy controls. The miRNAs expression levels were log2-transformed.

For each one among 842 human miRNAs encoded within three digits, we compared the miRNA expression levels of the ESCC patients and the normal controls. By controlling the FDR and FWER at 0.05, we identified seven significantly differentially expressed miRNAs, including miR-16-5a, miR-92-3a, miR-107, miR-320C, miR-451a, miR-486, and miR-574 (Table 2). The heatmap of the seven differential miRNAs indicated that their expression levels were consistent within each group but obviously different between the two groups (Figure 2). In addition, as shown in Figure 3, the serum levels of these identified miRNAs were all significantly up-regulated in ESCC patients than in healthy controls.

**Table 2:** The seven selected miRNAs by controlling FDR and FWER.

**miRNA**	**Log**_****2****_ **of expression level (mean ± standard deviation)**		***p*-value**	**Adjusted** ***p*****-value**	
	**ESCC (*n*=52)**	**Healthy *(n*=52)**		**FDR**	**FWER**
miR-451a	6.74 ± 2.72	4.08 ± 2.27	1.29 × 10^−6^	0.001	0.001
miR-16-5p	3.77 ± 2.42	1.57 ± 1.63	6.33 × 10^−6^	0.003	0.005
miR-486-5p	4.06 ± 2.59	1.90 ± 1.84	4.15 × 10^−5^	0.009	0.035
miR-574-5p	4.23 ± 2.11	2.74 ± 1.69	4.15 × 10^−5^	0.009	0.035
miR-92a-3p	3.46 ± 2.21	1.51 ± 1.85	5.80 × 10^−5^	0.010	0.049
miR-107	1.67 ± 2.97	0.03 ± 0.38	2.62 × 10^−5^	0.037	0.211
miR-320c	2.96 ± 2.44	1.20 ± 1.55	3.81 × 10^−4^	0.046	0.319

*FDR, false discovery rate; FWER, family-wise error rate*.

### Selection of Significant MiRNAs by Lasso Logistic Regression

We also applied Lasso logistic regression to screen these 842 human miRNAs encoded within three digits. The regularization parameter in Lasso logistic regression was selected by performing the 10-fold cross-validation, in which the cross-validated binomial deviance and the misclassification error were used as the criteria of predictive performance (Supplementary Figure S1). In the cross-validation process, the array data were randomly split into 10 subsets. Because the selected miRNAs were sometimes slightly different among each analysis, we carried out the cross-validation for 100 times and then identified candidate miRNAs that were selected for more than 30 times out of the 100 cycles of cross-validation. To obtain a more manageable set of miRNAs, we sacrificed one more cross-validation error and finally identified six miRNAs, including miR-7b-5p, miR-107, miR-16-5p, miR-191-3p, miR-451a, and miR-574-5p, which all had a selection frequency higher than 90%.

### Identification of Significant MiRNAs With Penalized SVM

The SVM method is one of the most powerful classification techniques, which is widely used for analyzing microarray data. Since the standard SVM cannot automatically select significant genes, to identify candidate miRNA biomarkers we performed the HHSVM and the SESVM. In these two methods, the tuning parameters that control the elastic-net penalty were selected by performing multiple rounds of 5-fold cross-validation. The margin-based loss function and misclassification error (ME) were employed for controlling cross-validation errors (Supplementary Figures S2, S3) for the HHSVM and the SESVM. We iterated the cross-validation 100 times for the high-dimensional microarray datasets and computed the selection frequency of each miRNA. MiRNAs with selection frequency of more than 30% were selected as significant ones. The HHSVM and the SESVM identified the same set of six miRNAs as Lasso logistic regression, including miR-7b-5p, miR-107, miR-16-5p, miR-191-3p, miR-451a, and miR-574-5p. The selection frequencies of these six miRNAs by HHSVM and SESVM methods using two types of cross-validation errors were summarized in Table 3. As shown in Table 3, five miRNAs were selected for more than 80 times out of 100 random splits by HHSVM and SESVM.

**Table 3:** The selection frequencies (%) of candidate miRNAs.

**miRNA**	**Lasso LR**		**HHSVM**		**SESVM**	
	**BD**	**ME**	**MBL**	**ME**	**MBL**	**ME**
let-7b-5p	95	92	67	73	58	69
miR-107	99	96	94	90	91	81
miR-16-5p	100	99	100	96	100	96
miR-191-3p	99	96	96	90	91	86
miR-451a	100	100	100	100	100	100
miR-574-5p	99	98	98	90	93	88

*Lasso LR, Lasso logistic regression; HHSVM, the hybrid Huberized support vector machine; SESVM, support vector machine using squared-error loss; BD, binomial deviance; ME, misclassification error; MBL, marginal based loss*.

### The Diagnostic Values of Selected Serum MiRNAs in ESCC

Notably, four miRNAs, miR-107, miR-16-5p, miR-451a, and miR-574-5p were simultaneously identified to be differentially expressed between ESCC and controls with all feature selection methods performed in this study. Although miR-107 was significant when the FDR was controlled at a level of 0.05, it was not significant if the FWER was also controlled at the same level. Furthermore, miR-107 had two outliers with extremely high levels, resulting in very large standard deviances in both ESCC and control groups. Therefore, miR-107 was excluded from further analysis and only the remaining three miRNAs, miR-16-5p, miR-451a, and miR-574-5p, were defined as potential biomarkers for the diagnosis of ESCC.

**Figure 4:**
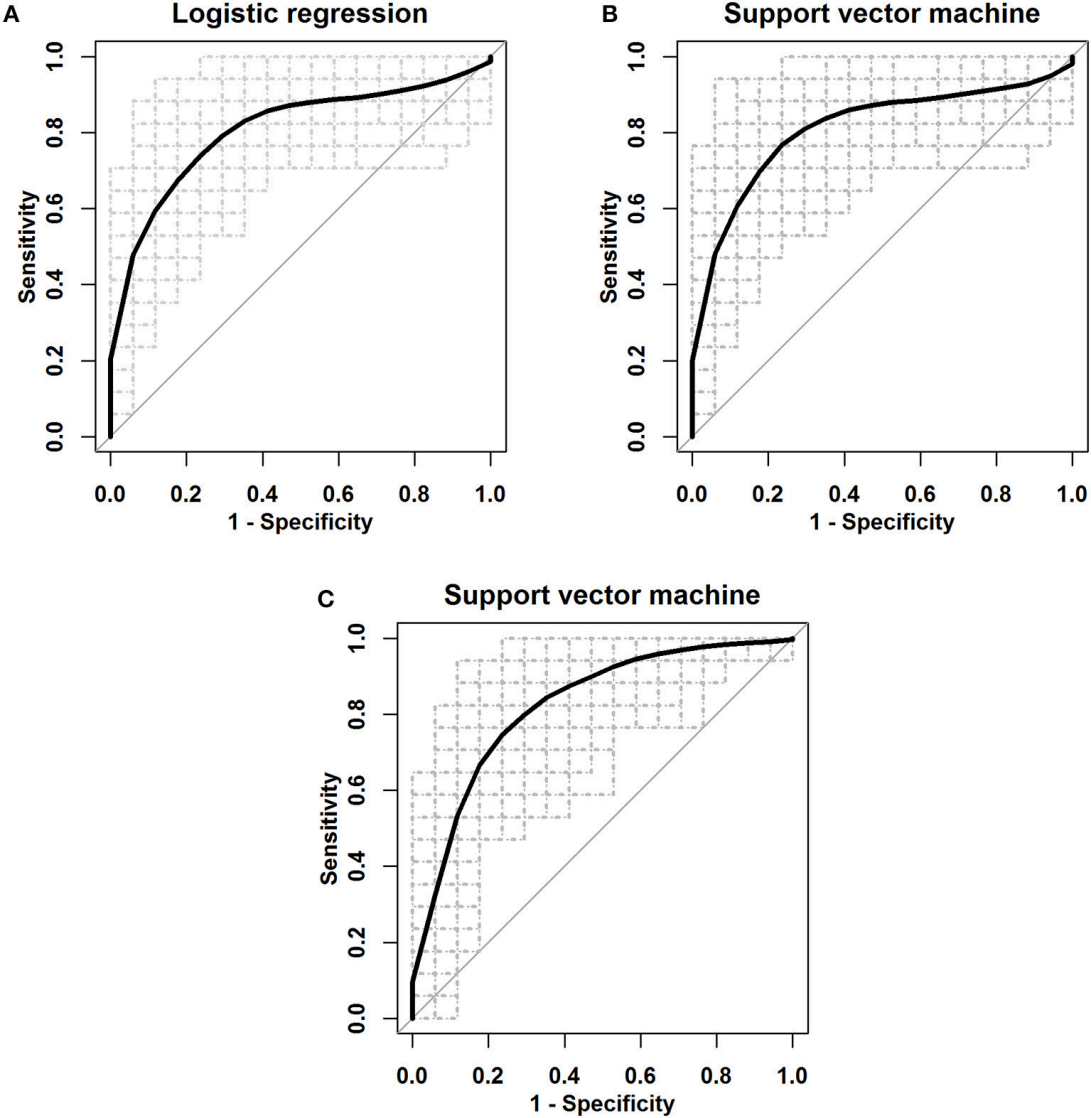
The ROC curves for the three serum miRNA-based panel as a diagnostic biomarker by using three classifiers. **(A)** Logistic regression. **(B)** Linear SVM. **(C)** SVM with the Radial Basis Function kernel.

To evaluate the efficiency of this novel panel of three biomarkers in the detection of ESCC, logistic regression and SVM were applied to develop three-miRNA-based classifiers for predicting ESCC. We also used the panel of the three significant miRNAs and the main clinical characteristics including age, sex, smoking status, and drinking status for the classifiers. We randomly split the miRNA profile data into the training and the testing sets 100 times. In each split, 35 ESCC patients and 35 healthy controls were randomly classified into the training group and the rest 17 ESCC and 17 controls were correspondingly defined as the testing group; the ROC curves were plotted and the AUCs were calculated with logistic regression, linear SVM and SVM with the Radial Basis Function kernel (Figure 4 and Supplementary Figure S4). As shown in Table 4, the average AUCs and average accuracies of the three classifiers with the significant three miRNAs all exceeded 0.80 and 0.79, respectively; the average sensitivities and specificities of the two methods both were larger than 0.74. Furthermore, the diagnostic performance of the three-miRNA-based classifiers was very similar to (a little worse than in some settings) that of the classifiers with the panel of three significant miRNAs and the main clinical characteristics. These results indicated that the panel of three miRNAs had high predictive accuracy for distinguishing ESCC from the healthy group with relatively high sensitivity and specificity.

**Table 4:** Diagnostic performance in discriminating ESCC from the healthy.

**Method**	**AUC (95% CI)**	**Sensitivity (SD)**	**Specificity (SD)**	**Accuracy (SD)**
**CLASSIFIER WITH THE THREE miRNAs**				
Logistic regression	0.812 (0.704, 0.905)	0.807 (0.110)	0.791 (0.117)	0.799 (0.047)
Linear SVM	0.816 (0.704, 0.907)	0.814 (0.104)	0.807 (0.105)	0.811 (0.046)
Radial SVM	0.824 (0.696, 0.927)	0.857 (0.096)	0.746 (0.110)	0.802 (0.049)
**CLASSIFIER WITH THREE miRNAs AND SEX, AGE, SMOKING, AND DRINKING**				
Logistic regression	0.821 (0.685, 0.926)	0.822 (0.120)	0.787 (0.134)	0.804 (0.057)
Linear SVM	0.832 (0.706, 0.921)	0.849 (0.113)	0.774 (0.134)	0.811 (0.051)
Radial SVM	0.836 (0.686, 0.929)	0.836 (0.111)	0.779 (0.136)	0.808 (0.053)

*AUC, area under the curve; CI, confidence interval; SD, standard deviation; SVM, support vector machine; Radial SVM, SVM with the Radial Basis Function kernel*.

**Figure 5:**
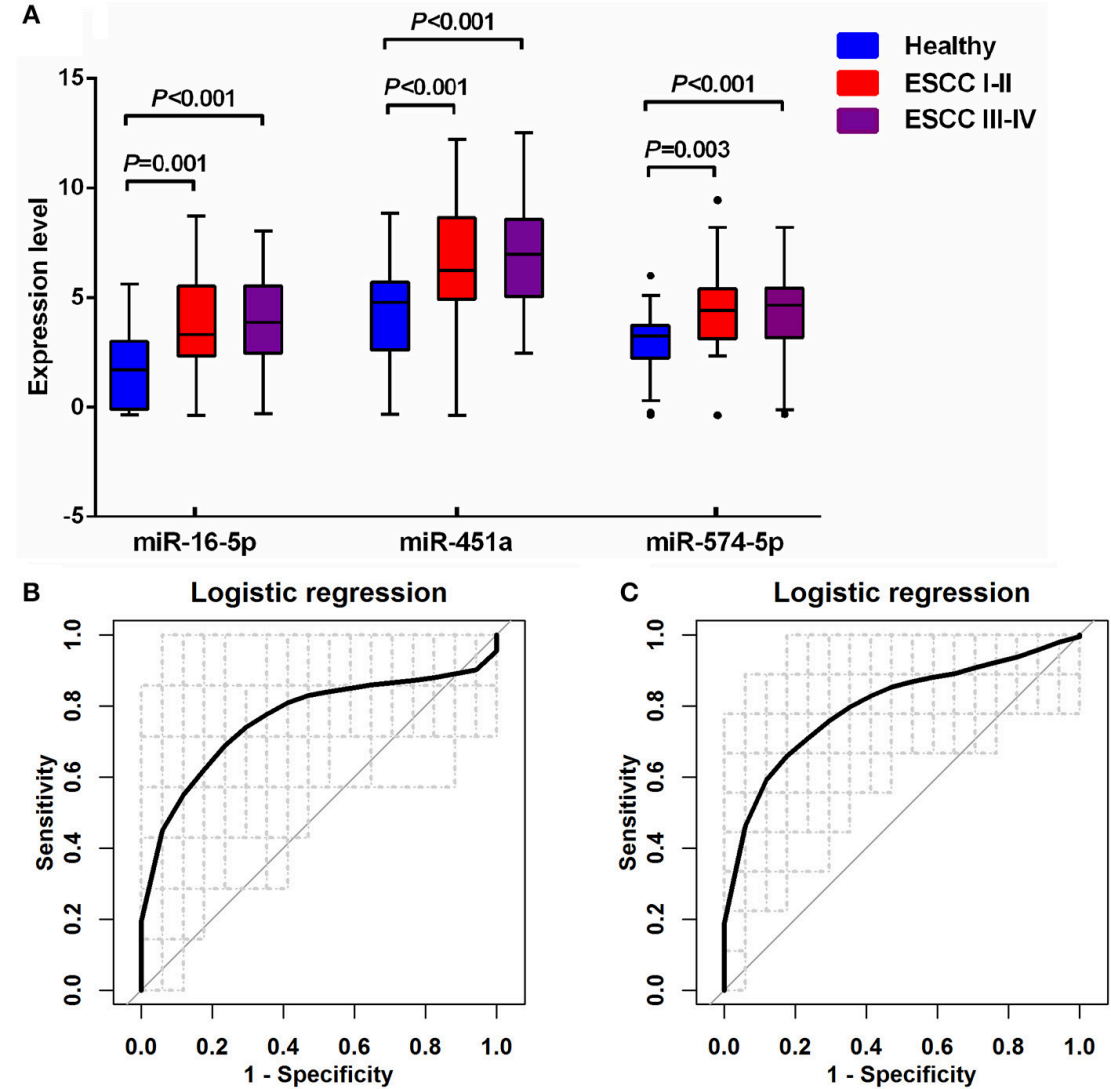
Quantification of serum miR-16-5p, miR-451a, and miR-574-5p in the healthy controls and two different ESCC groups. **(A)** The box plot comparing the expression level of serum miR-16-5p, miR-451a, and miR-574-5p in the healthy control with ESCC patients with stage I-II (ESCC I-II) and with stage III-IV (ESCC III-IV). **(B)** The ROC curve for the three serum miRNA-based panel in differentiating ESCC I-II from the healthy by logistic regression. **(C)** The ROC curve for the three serum miRNA-based panel in differentiating ESCC III-IV from the healthy by logistic regression.

Multiple comparisons of three miRNA levels between patients with ESCC stage I-II (ESCC I-II), ESCC stage III-IV (ESCC III-IV) and the healthy groups were further performed (Figure 5A and Supplementary Table S1). In both patient subgroups, the levels of miR-16-5p, miR-451a, and miR-574-5p were significantly increased compared with the healthy group (*p* < 0.005 for all three miRNAs). The diagnostic performance of the miRNA panel in different ESCC stages was further evaluated by using 100 times cross-validation (Table 5, Figures 5B,C, and Supplementary Figure S5). The analysis demonstrated that the miRNA panel had high accuracy in discriminating the healthy group from patients with ESCC stage I-II (AUC > 0.76, sensitivity > 0.73, and specificity > 0.82) and patients with ESCC stage III-IV (AUC > 0.80, sensitivity > 0.75, and specificity > 0.81). The similar (slightly better in some settings) results of diagnostic accuracy were obtained by adding the main characteristics in the three-miRNA-based classifiers (Supplementary Table S2). This indicated that the diagnostic performance of the miRNA panel was independent of ESCC status. Thus, the expression levels of miR-16-5p, miR-451a, and miR-574-5p panel can potentially serve as an efficient diagnostic biomarker for ESCC.

**Table 5:** Diagnostic performance in differentiating ESCC subgroups from the healthy for the three-miRNA-based classifiers.

**Method**	**AUC (95% CI)**	**Sensitivity (SD)**	**Specificity (SD)**	**Accuracy (SD)**
**DIFFERENTIATING THE ESCC I-II FROM THE HEALTHY**				
Logistic regression	0.769 (0.563, 0.971)	0.756 (0.165)	0.839 (0.126)	0.781 (0.107)
Linear SVM	0.787 (0.613, 0.979)	0.791 (0.140)	0.828 (0.130)	0.803 (0.091)
Radial SVM	0.779 (0.617, 0.971)	0.737 (0.171)	0.821 (0.156)	0.763 (0.096)
**DIFFERENTIATING THE ESCC III-IV FROM THE HEALTHY**				
Logistic regression	0.805 (0.633, 0.938)	0.752 (0.153)	0.845 (0.122)	0.786 (0.078)
Linear SVM	0.801 (0.617, 0.951)	0.794 (0.134)	0.839 (0.093)	0.811 (0.076)
Radial SVM	0.816 (0.683, 0.942)	0.793 (0.119)	0.815 (0.095)	0.801 (0.066)

*AUC, area under the curve; CI, confidence interval; SD, standard deviation; SVM, support vector machine; Radial SVM, SVM with the Radial Basis Function kernel*.

## Discussion

In this study, we systematically investigated the serum miRNA profiles of a relatively large cohort of 52 ESCC patients and 52 well-matched healthy controls using high-throughput microarray-based assay. Using the FDR method, the FWER method, Lasso Logistic regression, HHSVM and SESVM together, this study has identified three miRNAs, miR-16-5p, miR-451a, and miR-574-5p, as potential biomarkers for diagnosis of ESCC. The expression levels of these three miRNAs were significantly up-regulated in the serum of ESCC patients compared to normal controls. We developed classifiers based on these three miRNAs with the logistic regression and the SVM model to predict ESCC. The classification accuracy and AUC were used to assess the performance of the three miRNAs as a diagnostic tool in detecting ESCC. The average AUCs and classification accuracies based on different classifiers were higher than 0.80 and 0.79, respectively, indicating that the three-miRNA-based panel had a high potential to distinguish ESCC patients from healthy controls.

This study has major strengths. To the best of our knowledge, this is the first study in the identification of potential serum miRNAs as diagnostic biomarkers in ESCC based on microarray data of exceeding 100 sample cases, which increased the statistical power of each test and had a stronger sensitivity for quantitative detection of the serum miRNAs. Furthermore, five representative feature selection algorithms, which are appropriate for high dimension and low sample data analysis, were together performed to select significant miRNAs with a high credibility. Lastly, reliable validation employing 100 times random splits for the samples has shown that miRNA-based classifiers were powerful for the diagnosis of ESCC.

The biological functions of the three miRNAs identified in our study have been investigated in previous studies. MiR-16 was one of the earliest miRNAs found to be involved in cancers; it could suppress apoptosis and promote cell growth by down-regulating reversion-inducing-cysteine-rich protein with Kazal motifs (RECK) and sex-determining region Y-box (Sox) 6, two genes that play important roles in the pathogenesis of ESCC (18). MiR-451 is a key factor in regulating erythroid differentiation and in maintaining homeostasis of erythroid cells (19). It has also been associated with cell proliferation, migration and apoptosis via regulating different target genes (20), such as calcium-binding protein 39 (CAB39) (21), Ras-related protein 14 (RAB14) (22), and macrophage migration inhibitory factor (MIF) (23). MiR-574-5p has been reported to act as an oncogene in various types of cancers, including ESCC (24, 25).

There are some limitations of this study that need to be declared. First, the microarray experiment in this study was not designed specifically to identify biomarkers distinguishing between ESCC and normal esophagus, but rigorous dimensionality reduction techniques were employed to strengthen its effect of screening miRNAs. Second, although our study design is a population-based case-control study, potential drawbacks such as selection bias may still occur, because the ESCC cases were selected from the Cancer Hospital of Linzhou City, which accounted for above 60% of the esophageal cancer patients in the study population. However, the healthy controls were recruited from the general population in Linzhou City. Moreover, this case-control study design is not warranted to infer causal relationships between the expression levels of miRNAs and ESCC. Third, the levels of the three candidate miRNAs selected from the microarray were not further quantified by quantitative real-time PCR (qRT-PCR). However, miRNA microarray expression has shown to be highly concordant when re-analyzed with qRT-PCR (26), with correlation coefficients measuring from *r* = 0.986 to 0.994, depending on normalization method (27). Further validation of the newly identified miRNAs in larger independent population is necessary before they can be put in a clinical application.

## Conclusion

In summary, five useful feature selection algorithms were jointly adopted to select the common serum miRNA signatures for the diagnosis of ESCC based on microarray data. Three serum miRNAs were identified, and classifiers based on the combination of them were constructed by the Logistic regression and the SVM. The cross-validation results showed that the three-miRNA-based classifiers can accurately distinguish ESCC patients from healthy controls. This study provides a resource and an impetus for further investigating these novel serum miRNAs as biomarkers in ESCC.

## Data Availability

The miRNA datasets analyzed for this study can be found in the Gene Expression Omnibus (GEO) repository (http://www.ncbi.nlm.nih.gov/geo/query/acc.cgi?acc=GSE112840).

## Author Contributions

DZ, YH, WW, and FL conceived the study. YD and QM performed and evaluated the experiments. YS helped to perform the experiments. XG helped to collect the samples. DZ, YD, and LZ analyzed the data. DZ, LZ, and FL wrote the manuscript. All authors contributed to the preparation of the manuscript. All authors read and approved the final manuscript.

### Conflict of Interest Statement

The authors declare that the research was conducted in the absence of any commercial or financial relationships that could be construed as a potential conflict of interest.
