# Supplementary material for: Identification of Serum MicroRNAs as Novel Biomarkers in Esophageal Squamous Cell Carcinoma Using Feature Selection Algorithms
Source: Front Oncol. 2019 Jan 21;8:674. doi: 10.3389/fonc.2018.00674 (PMC6348251; doi:10.3389/fonc.2018.00674)
Supplement: Supplementary file 1 [file Data_Sheet_1.docx]

***Supplementary Material***

**Identification of Serum MicroRNAs as Novel Biomarkers in Esophageal Squamous Cell Carcinoma Using Feature Selection Algorithms**

**Deqiang Zheng^1¶^, Yuanjie Ding^1¶^, Qing Ma^2^, Lei Zhao^3^, Xudong Guo^1^, Yi Shen^1^, Yan He^1^,**

**Wenqiang Wei^2*^, Fen Liu^1*^**

*** Correspondence:** Fen Liu: liufen05@ccmu.edu.cn; Wenqiang Wei: weiwq@cicams.ac.cn

# Supplementary Figures and Tables

## Supplementary Figures

##
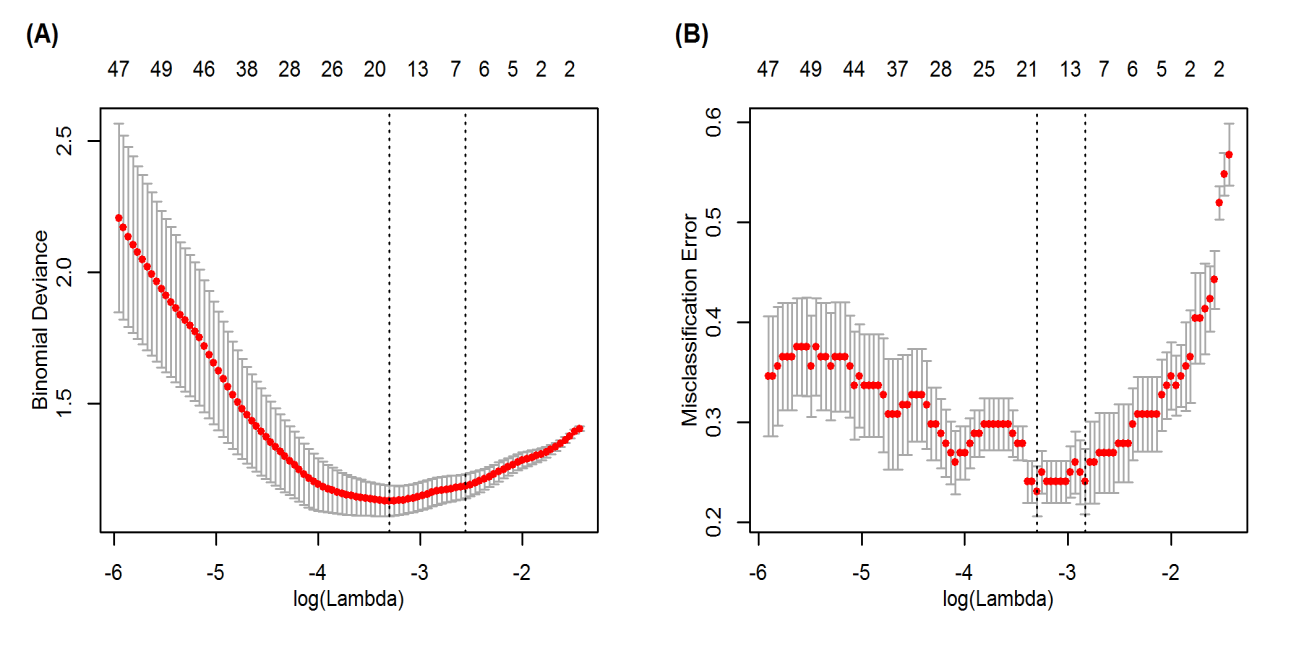


**Supplementary Figure S1.** The cross-validation curves for selection of tuning parameters in the Lasso logistic regression. (A) Cross-validation using the binomial deviances. (B) Cross-validation using the misclassification error.


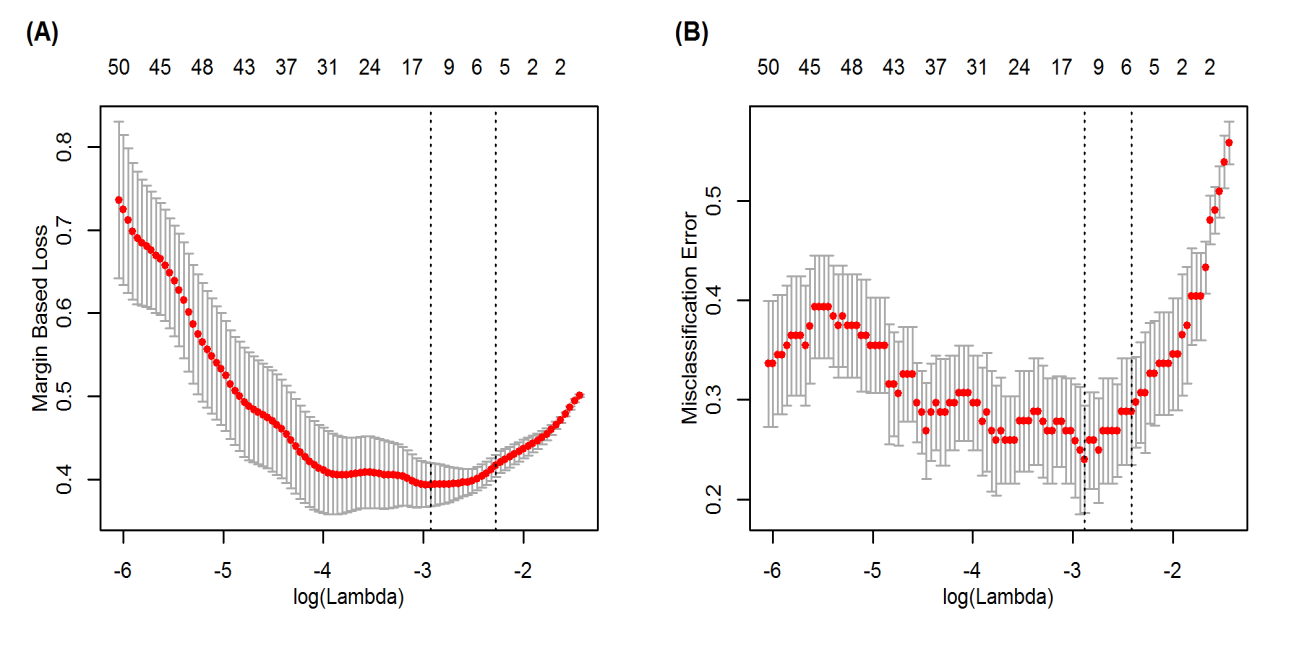


**Supplementary Figure S2.** The cross-validation curves for selection of tuning parameters in the hybrid huberized support vector machine. (A) Cross-validation using the margin based loss.

(B) Cross-validation using the misclassification error.


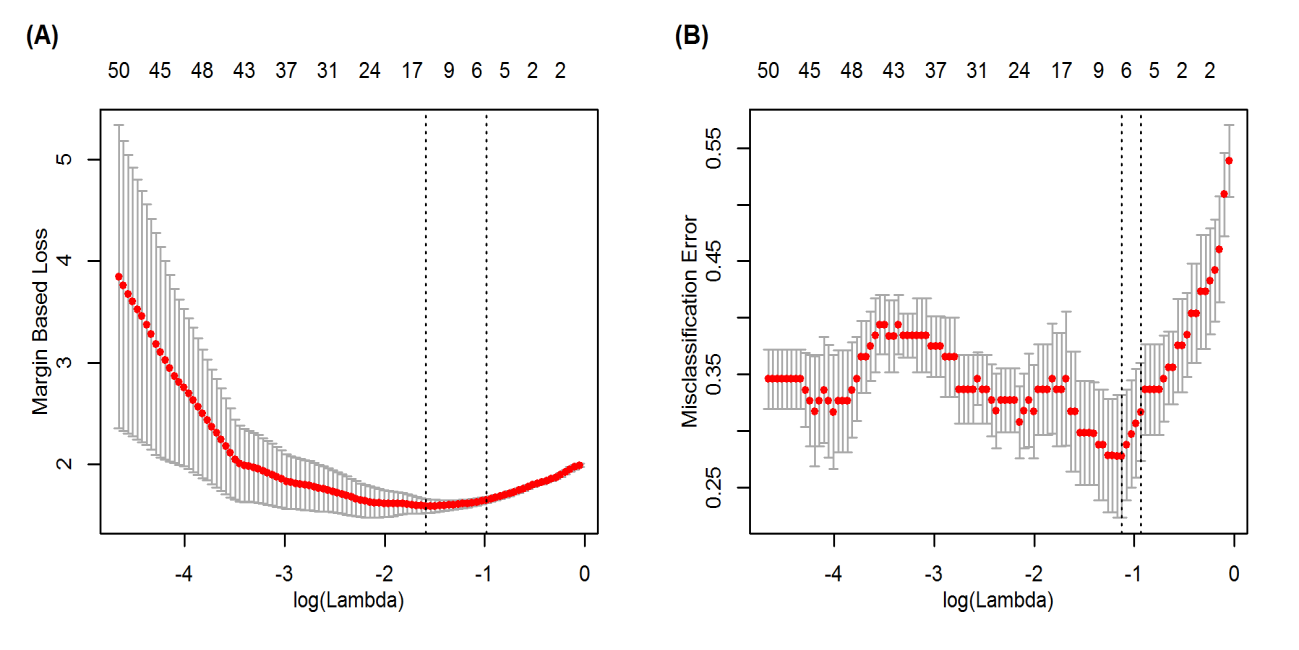


**Supplementary Figure S3.** The cross-validation curves for selection of tuning parameters in the support vector machine using the squared-error loss. **(A)** Cross-validation using the margin based loss. **(B)** Cross-validation using the misclassification error.


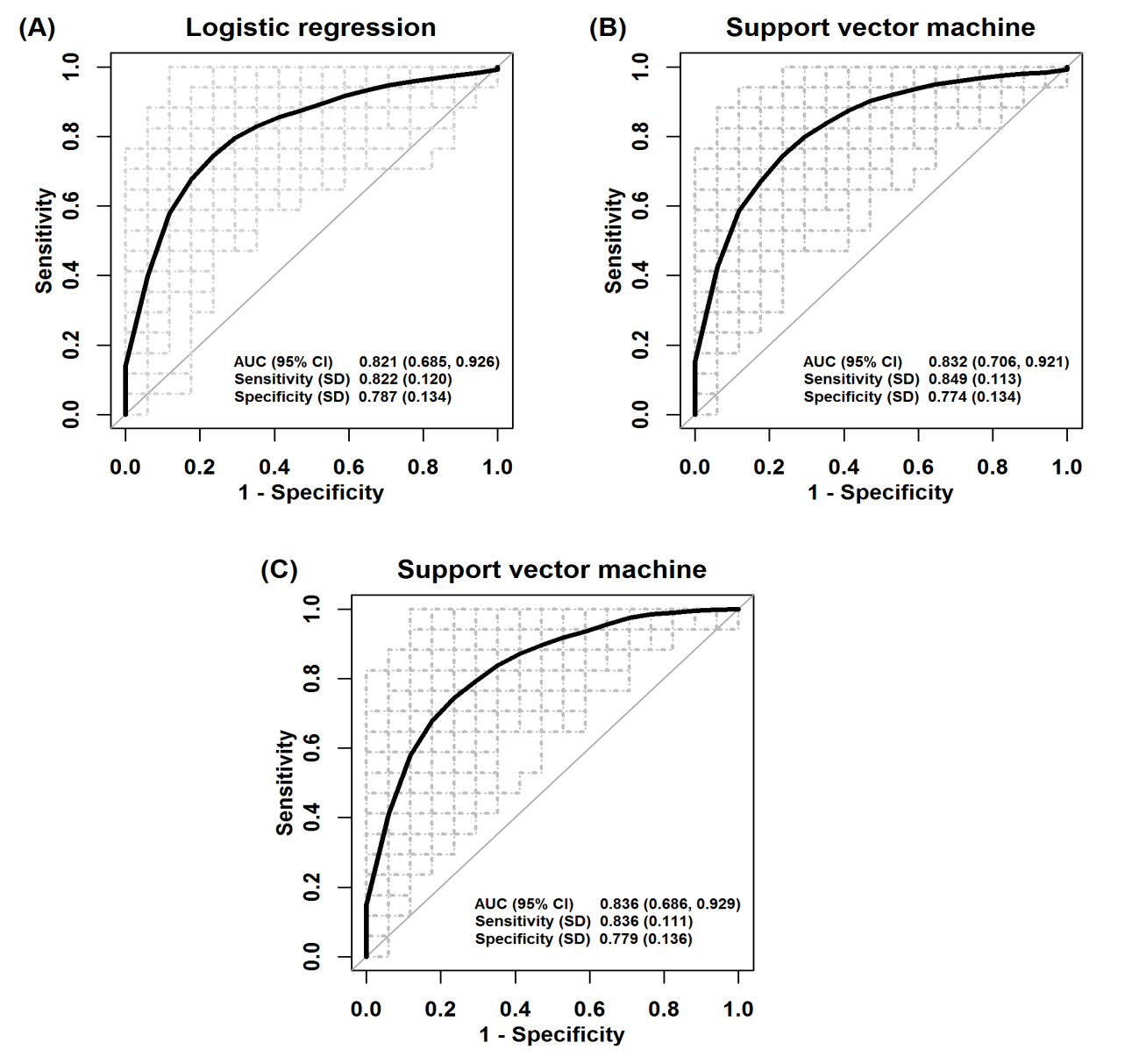


**Supplementary Figure S4.** The ROC curves for three classifiers with three serum miRNA-based panel and sex, age, smoking and drinking. **(A)** Logistic regression. **(B)** Linear SVM. **(C)** SVM with the Radial Basis Function kernel.


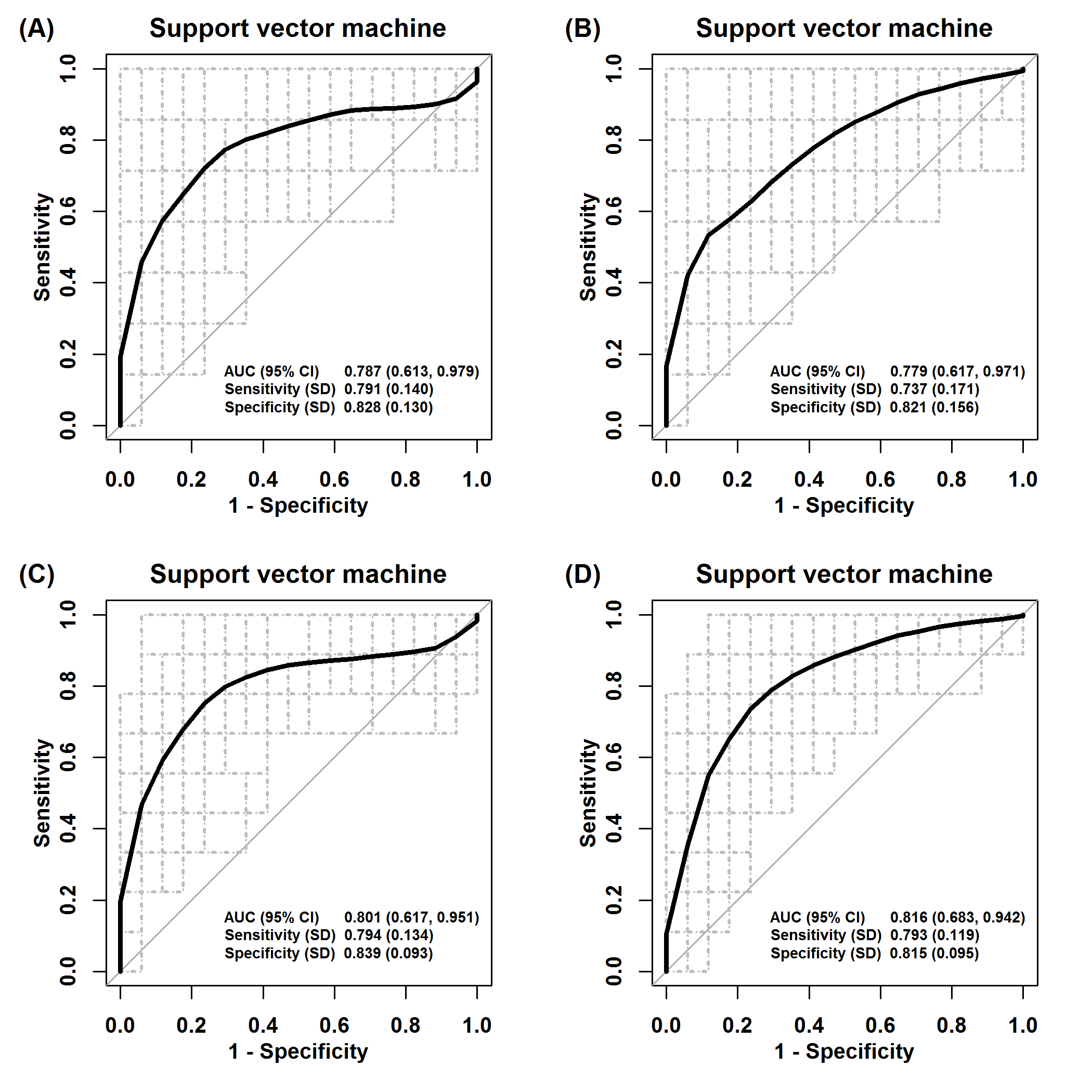


**Supplementary Figure S5**. The ROC curves for three classifiers with three serum miRNA-based panel. **(A)** Linear SVM for differentiating the ESCC I-II from the healthy. **(B)** SVM with the Radial Basis Function kernel for differentiating the ESCC I-II from the healthy. **(C)** Linear SVM for differentiating the ESCC III-IV from the healthy. **(D)** SVM with the Radial Basis Function kernel for differentiating the ESCC III-IV from the healthy.

## Supplementary Tables

**Supplementary Table S1** | Multiple comparisons of the expression profiles of

three significant miRNAs in two different ESCC groups and the healthy group

| **miRNA** | **Log2 of expression level**  **(mean ± standard deviation)** | | |  | **^a^*p*-value** | **^b^*p*-value** | **^c^*p*-value** |
| --- | --- | --- | --- | --- | --- | --- | --- |
|  | **Healthy**  **(*n*=52)** | **ESCC I-II (*n*=23)** | **ESCC III-IV (*n*=29)** |  |  |  |  |
| miR-16-5p | 1.57±1.63 | 3.70±2.61 | 3.82±2.30 |  | 0.001 | <0.001 | 0.798 |
| miR-451a | 4.08±2.27 | 6.58±3.15 | 6.87±2.38 |  | <0.001 | <0.001 | 0.913 |
| miR-574-5p | 2.74±1.69 | 4.32±2.29 | 4.16±1.99 |  | 0.003 | <0.001 | 0.898 |

ESCC I-II: ESCC with stage I-II; ESCC III-IV: ESCC with stage III-IV; ^a^: ESCC I-II versus the

healthy. ^b^: ESCC III-IV versus the healthy. ^c^: ESCC I-II versus ESCC III-IV.

**Supplementary Table S2** | Diagnostic performance in differentiating ESCC subgroups

from the healthy for classifiers with the panel of three miRNAs and characteristics

| **Method** | **AUC (95% CI)** | **Sensitivity (SD)** | **Specificity (SD)** | **Accuracy (SD)** |
| --- | --- | --- | --- | --- |
| **Differentiating the ESCC I-II from the healthy** | | |  |  |
| Logistic regression | 0.734 (0.495, 0.954) | 0.729 (0.159) | 0.809 (0.159) | 0.753 (0.103) |
| Linear SVM | 0.796 (0.639, 0.972) | 0.797 (0.145) | 0.822 (0.128) | 0.805 (0.094) |
| Radial SVM | 0.781 (0.580, 0.950) | 0.809 (0.164) | 0.751 (0.159) | 0.791 (0.091) |
| **Differentiating the ESCC III-IV from the healthy** | | | |  |
| Logistic regression | 0.852 (0.699, 0.971) | 0.846 (0.149) | 0.821 (0.136) | 0.837 (0.079) |
| Linear SVM | 0.843 (0.706, 0.951) | 0.848 (0.136) | 0.805 (0.120) | 0.832 (0.072) |
| Radial SVM | 0.869 (0.768, 0.967) | 0.878 (0.105) | 0.828 (0.100) | 0.860 (0.059) |

AUC: area under the curve; CI: confidence interval; SD: standard deviation; SVM: support vector machine;

Radial SVM: SVM with the Radial Basis Function kernel.
